# No evidence of a cleaning mutualism between burying beetles and their phoretic mites

**DOI:** 10.1038/s41598-017-14201-6

**Published:** 2017-10-23

**Authors:** Ana Duarte, Sheena C. Cotter, Ornela De Gasperin, Thomas M. Houslay, Giuseppe Boncoraglio, Martin Welch, Rebecca M. Kilner

**Affiliations:** 1 Department of Zoology, University of Cambridge, Downing Street, Cambridge, CB2 3EJ U.K.; 2 University of Exeter, Penryn, TR10 9FE U.K.; 3 School of Life Sciences, University of Lincoln, Brayford Pool, Lincoln, LN6 7TS U.K.; 4 Department of Ecology and Evolution, University of Lausanne, 1015 Lausanne, Switzerland; 5 Department of Biochemistry, University of Cambridge, Cambridge, CB2 1QW U.K.

## Abstract

Burying beetles (*Nicrophorus vespilloides*) breed on small vertebrate carcasses, which they shave and smear with antimicrobial exudates. Producing antimicrobials imposes a fitness cost on burying beetles, which rises with the potency of the antimicrobial defence. Burying beetles also carry phoretic mites (*Poecilochirus carabi* complex), which breed alongside them on the carcass. Here we test the novel hypothesis that *P*. *carabi* mites assist burying beetles in clearing the carcass of bacteria as a side-effect of grazing on the carrion. We manipulated the bacterial environment on carcasses and measured the effect on the beetle in the presence and absence of mites. With next-generation sequencing, we investigated how mites influence the bacterial communities on the carcass. We show that mites: 1) cause beetles to reduce the antibacterial activity of their exudates but 2) there are no consistent fitness benefits of breeding alongside mites. We also find that mites increase bacterial diversity and richness on the carcass, but do not reduce bacterial abundance. The current evidence does not support a cleaning mutualism between burying beetles and P. carabi mites, but more work is needed to understand the functional significance and fitness consequences for the beetle of mite-associated changes to the bacterial community on the carcass.

## Introduction

Interactions between species, ranging from competition to mutualism, are a key driver of biodiversity. The outcome of such interactions for the fitness of individuals in a population can vary with individual characteristics and environmental conditions^1,2^. These fitness consequences influence not only the co-evolution of traits mediating interspecific interactions^3^, but also the evolution of life-history and social behavioral traits^4,5^, by changing the adaptive landscape in which these traits evolve.

Host-parasite interactions have been particularly well-studied in the context of parental care, and are implicated in the trade-off between current and future reproduction^6^. Parasites may decrease the value of the current brood. In great tits, for example, flea infestations decreased nestling mass and number^7^, and led to reduced brooding and nestling care^8^. At the other end of the spectrum, mutualistic interactions may reduce the costs of parental care. For example, in an ant-treehopper mutualism, attendance by ants frees the female treehopper to leave their first clutch of eggs in the ants’ care, to produce new clutches herself^9^. The female treehopper therefore transfers some of the costs of parental care to the mutualistic partner.

Here we examine how interspecific interactions modulate a parental investment trait in the burying beetle, *Nicrophorus vespilloides*: the lytic activity of its anal exudates. Burying beetles use small vertebrate carcasses to rear their offspring; they prepare the carcasses for breeding by shaving them, rolling them into a ball, smearing them with antimicrobial exudates and burying them in a shallow grave^10–12^. The eggs are laid in the surrounding soil, and larvae hatch within 3–4 days. Both parents can feed the larvae with regurgitated meat from the carcass, but males typically desert the brood earlier than females^13,14^. Larvae feed for approximately 4–5 days, then disperse from the remains of the carcass to pupate in the soil.

Burying beetles carry with them several species of phoretic mites^15,16^. The association with phoretic mites has occasional short-term benefits for beetle fitness, because mites eliminate blowfly larvae; the presence of mites may also have long-term positive effects for beetles, due to a reduction in the number of nematodes carried by beetles^17^. We focus on the association between *N*. *vespilloides* and the *Poecilochirus carabi* species complex. *P*. *carabi* (hereafter ‘mites’) attach to burying beetles as deutonymphs (juveniles) when the beetles breed or feed on carcasses. Burying beetles typically carry approximately ten mites, but individuals carrying up to hundreds have been observed in the field^15,18^. Nevertheless, burying beetles make no attempt to self-groom or remove mites. Mites seemingly derive no nourishment from the beetle, and use it simply as a means of transport^15^. When adult beetles locate a carcass, the mites alight, feed on the carcass, molt into adults, mate, and reproduce on the carrion, living alongside the beetle larvae on the carrion. It is during reproduction on the carcass that mites are most likely to influence burying beetle fitness. When the parents depart at the end of reproduction, they carry with them the next generation of mites^15^.

Previous work has found that the effect of mites on beetle fitness varies with sex and ecological conditions^14,19^. Mites can increase the costs of pre-hatching care (i.e. carcass preparation) for *N*. *vespilloides* males^19^. Males desert the brood earlier when mites are present^14^ and in doing so avoid fitness costs in terms of lifespan and decreased brood size. Female desertion time, however, is not affected by mites, even though the earlier the male leaves, the more mites are carried by the female. There are two potential reasons for why males and females evolved different responses to the presence of mites. First, females may be constrained on departure time, because the earlier the female leaves, the smaller is the surviving brood^14^. Hence females may simply be making the best of a bad job. A second potential reason is asymmetric investment in different components of parental care by each sex.

One component of care which differs between male and female beetles is the antibacterial defence of the carcass, with female exudates showing stronger lytic activity than males^12^. This lytic activity is part of the beetle’s social immune system (*sensu* Cotter and Kilner 2010^20^) because it potentially protects adult beetles and larvae from pathogenic microbes on the carcass. However, mounting this response imposes a fitness cost on females^21^. It also increases larval survival^22^, and can therefore be considered an integral part of parental investment in *N*. *vespilloides*. We test the hypothesis that mites modulate the costs to female burying beetles of defending the carrion with antimicrobials. Mites could achieve this by grazing on the surface of the carrion, thereby ingesting bacteria and fungi. Mites may also produce their own antimicrobial defences, which are common in invertebrates that breed on microbially rich resources (e.g. *Nicrophorus*, blowflies, houseflies^23,24^. Both of these behaviours are likely to evolve as part of the mites’ carrion-feeding ecology. The consequent reduction in costs of antimicrobial defence to the burying beetle host would be a by-product of natural selection on mites to selfishly exploit the transport to carrion on the beetle. Nevertheless, this could explain why females (who invest more in this trait) may tolerate, and even benefit under some circumstances, from mites, whereas males do not.

We investigated whether mites are in a by-product cleaning mutualism with burying beetles. Specifically, we asked: do female beetles benefit from the mites because they clear the carcass of microbes, and consequently reduce the costs of antimicrobial defence? We tested this idea in two ways. First we investigated whether mite presence reduces the lytic activity of the female’s anal exudates, and thereby reduces the fitness costs associated with antimicrobial defence of the carcass. In this experiment, we manipulated the bacterial community on the carcass and measured a female’s lytic activity and components of fitness in the presence and absence of mites. In a second experiment, we investigated the effect of mites on the bacterial communities growing on mouse carcasses prepared by beetles for reproduction. Using molecular approaches, including quantitative real-time PCR and culture-independent 16 S rDNA-based compositional analysis of bacterial communities, we measured the bacterial load (i.e. a proxy for number of bacterial cells) on the carcass, bacterial community richness and diversity, and community composition, on carcasses with and without mites.

## Methods

The experiments were carried out from January to June 2012. We used beetles from a laboratory stock population established in 2005 at the University of Cambridge from wild beetles caught in woodlands surrounding Cambridge. Every summer, field-caught beetles were added to the laboratory stock to maintain genetic diversity. Maintenance of the laboratory stock is described in detail elsewhere^21^. In brief, the stock population was kept under standard conditions of temperature and photoperiod. Adult beetles were maintained individually in plastic boxes filled with moist soil and fed with minced beef twice a week. Sexually mature males and females (12–15 days after eclosion) were paired in plastic containers half-filled with moist soil and were provided with a thawed mouse carcass (12–16 g). Breeding pairs were kept in darkness to simulate underground conditions. Larvae hatch 72 h after pairing males and females, complete their development on the carcass and start dispersing into the surrounding soil five days after hatching. Dispersing larvae were placed in plastic boxes of 5 × 5 individual divisions, covered with moist compost and left to pupate (approximately 3 weeks). The life-cycle of *N*. *vespilloides* therefore takes approximately 6 weeks under laboratory conditions. All mice carcasses used in this study were obtained frozen from LiveFoods Direct^TM^.

### Mite laboratory stock

The mite laboratory stock was established from deutonymphs (juvenile phoretic stage) collected from field-caught beetles in September 2011. Mites were removed using a brush and tweezers and transferred to plastic containers filled with moist soil, and a single burying beetle. They were fed minced beef once per week. Once per month, we bred mites by introducing approximately ten deutonymphs into a plastic container with moist soil and a dead mouse, and adding a pair of sexually mature burying beetles. Eight days later, when reproduction was complete, the next generation of deutonymphs was collected from the adult beetles. We anaesthetized adult beetles using CO_2_, removed mites with a brush and tweezers, and transferred them to plastic soil-lined containers.

#### Experiment 1: can mites reduce lytic activity and its associated fitness costs

Two aspects of the breeding conditions were manipulated, in a 2 × 2 balanced design: the bacterial environment in the carcass; and the presence or absence of phoretic mites. We manipulated the bacterial environment by dipping mouse carcasses in a bacterial suspension, which has been shown in previous work to lead to up-regulation of lytic activity of the anal exudates of breeding females^21^, without directly harming females. Half of the carcasses were dipped in a bacterial suspension of *Micrococcus luteus*. We used *M*. *luteus* because it is a common soil bacterium and is the standard microbe used in the assay of lytic activity. Furthermore, its presence has been demonstrated to upregulate lytic activity in *N*. *vespilloides* without direct effects on the beetle’s survival^21^. As a control, the remaining carcasses were dipped in a sterile nutrient broth. To test whether the presence of mites affects regulation of social immunity, we added ten deutonymphs of *P*. *carabi* to half of the bacterially-challenged carcasses and to half of the control-dipped carcasses. We therefore obtained four treatments: control-dipped without mites, control-dipped with mites, bacteria-dipped without mites, and bacteria-dipped with mites. The average carcass mass was 10.82 ± 1.68 g, and did not vary by treatment (ANOVA: *F*_3_ = 2.032, *p* = 0.108)

We paired 180 virgin, sexually matured females (2–3 weeks old), in three separate batches of 60, with 2–3 week old unrelated virgin, sexually matured males. The pairs were distributed between the four carcass treatments. Whenever possible, tetrads of sisters were assigned to the four treatments, allowing us to control for genetic factors. A pilot experiment (Figure S1 in Supplementary Material) indicated that when females were left to prepare the carcass alone, breeding success was lower in the presence of mites. We therefore allowed males to be present during carcass preparation, removing them just before larval hatching, at approximately 60 hours after pairing. This is within the range previously observed for male brood desertion in the presence and absence of mites^14^ (mean ± sd of male departure times from data presented in De Gasperin *et al*. 2015: 96.69 ± 45.67 h with mites; 114.41 ± 47.06 h without mites). Post-hatching care was performed exclusively by the female.

After 8 days of feeding on the carcass, larvae start to disperse. At this point, we counted and weighed the larvae. In mite-infested carcasses, any deutonymphs dispersing on the females were removed after larval dispersal; females were subjected to CO_2_ anesthesia, and deutonymphs were removed with a fine brush. Females in mite-free treatments also underwent CO_2_ anesthesia and were handled with a fine brush. After dispersal, females were maintained for five days under normal stock conditions, after which they were bred once more with the same manipulation of the breeding conditions. At the end of the second breeding event, females were cleaned of mites, as described above. Subsequently, the surviving females were allowed to breed on unmanipulated carcasses without mites until they died, with five days to rest between each breeding event. In every breeding event, each female was paired with a virgin male 2–3 weeks old; all males were removed prior to larval hatching. Female lifespan and male and female pronotum width, a reliable measure of individual size, were recorded. Males used for breeding events where carcass conditions were manipulated were also kept, under standard conditions, and their lifespan was recorded.

### Collection and analysis of anal exudates

In the first two breeding events, anal exudates were collected from females 72 h after pairing, when larvae start to hatch. Lytic activity peaks in the 24 h after larval hatching^25^, therefore making this a good point in time to assess female investment in social immunity. Female beetles readily produce anal exudates when gently tapped on the back of the abdomen. However, in some cases, females did not produce exudates (39 females in breeding 1 and 32 females in breeding 2). Exudates were collected in capillary tubes, stored in 1.5 ml Eppendorf tubes and kept frozen at −20 °C until further analysis. We performed lytic zone assays, following Cotter *et al*. 2010^21^, to calculate lytic activity, in mg per ml of lysozyme equivalents.

### Statistical analysis

Females that never produced offspring were excluded from all analyses. We used general linear mixed models to analyse lytic activity and reproductive output in the statistical programme R (package ‘lme4’^26^). Unless otherwise specified, *p*-values for lme4 models were calculated using the package ‘lmerTest’^27^, with denominator degrees of freedom calculated from Satterthwaite’s approximation. Lytic activity was log-transformed such that model residuals met the assumptions of normality for regression. Breeding failures were removed from the analysis of lytic activity. The measures of reproductive output, recorded at dispersal, were: brood size, brood mass, average larval mass and larval density (brood size divided by carcass mass). In all models regarding reproductive output, we initially included carcass mass (excluding larval density) and female pronotum width as covariates. For analysis of survival, we used mixed effects Cox proportional hazards models (package ‘coxme’^28^), with female pronotum width as a covariate. In most models we used a nested random structure, with female identity and female family (to account for variation due to genetic relatedness) nested in block. We applied model selection to find the minimal adequate model, following Zuur *et al*. (2009)^29^. Model selection was applied to models fitted with Maximum Likelihood (ML), and the minimal adequate model was then re-fitted with Restricted Maximum Likelihood (REML). All tables show minimal adequate models.

#### Experiment 2: do mites alter bacterial communities on the carcass

We repeated the manipulations to carcasses described above, obtaining again four treatments (*N* = 6 per treatment): control-dipped without mites, control-dipped with mites, bacteria-dipped without mites, and bacteria-dipped with mites. We randomly paired males and females from the stock and allowed each pair to prepare a carcass. When carcass preparation was complete (60 h after pairing) we removed the pairs and sampled the carcasses for bacterial DNA. The sampling protocol is described in detail in a previous study^30^. In brief, we washed carcasses in PBS to collect bacterial cells, pelleted the bacterial cells by centrifugation and kept pelleted material at −80 °C until DNA extraction. We isolated DNA using the FastDNA® Spin Kit for Soil (MP Bio Laboratories, Inc. Carlsbad, CA, USA), taking a volume of 750 µl of pelleted material from each sample to normalize the amount of bacterial DNA sampled. Quantitative real-time polymerase chain reaction (qRT-PCR) was performed on a fragment of the 16S rRNA-encoding gene (detailed methods in Supplementary Material), to assess bacterial abundance in the different treatments. Libraries for sequencing were prepared by an initial PCR-amplification of the full length bacterial 16S rRNA-encoding gene; PCR products were used in a second PCR, to amplify the V3 region of the 16S rRNA-encoding gene with Illumina-compatible primers. High-throughput paired-end sequencing was performed in an Illumina MiSeq instrument at the DNA Sequencing Facility (Department of Biochemistry, University of Cambridge). Sequence reads were de-noised and analyzed using MOTHUR v.1.35.1 (www.mothur.org) software package^31^, following the Standard Operating Procedure described in Kozich *et al*. (2013)^32^ and MOTHUR’s Wikipedia page (http://www.mothur.org/wiki/MiSeq_SOP, accessed August 2015). Full details are provided in the Supplementary Material. Sequences were clustered into operational taxonomic units (OTUs), using the average neighbour algorithm^33^ with a cut-off distance of 0.03. A consensus classification for each OTU was obtained. We generated a data matrix with every OTU and the number of reads belonging to each sample assigned to each OTU. To control for differences in the number of reads obtained per sample, we used a sub-sample of the dataset in all analyses.

### Statistical analysis

We tested for differences in bacterial DNA concentration between treatments, calculated by qPCR, with a general linear model. Community richness and diversity (inverse Simpson index) were analyzed with a general linear model, with mite exposure and carcass dipping treatment as factors. Differences in community composition were tested with PERMANOVA in R (package ‘vegan’^34^). The same model structure as the ANOVAs described above was used for PERMANOVA. Multivariate group dispersions (variances) were calculated with the package ‘vegan’ and an ANOVA was performed to test for multivariate homogeneity of variances.

To discern which bacterial groups may be affected by the presence of mites, we used Indicator Species Analysis in R^35^ to identify OTUs strongly associated with the occurrence of mites. Indicator Species Analysis is a standard community ecology approach taking into account both relative abundance (read numbers, in this case) and relative frequency of occurrence in various sites^36^. An OTU has maximal Indicator Value when all of its occurrences are found in a single site (i.e., treatment) and when it occurs in all instances of that group (i.e., all samples within a treatment).

### Data Availability

The datasets generated and analysed during the current study are available in the Cambridge Apollo repository (https://doi.org/10.17863/CAM.9284). DNA sequences are available in the NCBI Sequence Read Archive, BioProject RJNA384609.

## Results

### Experiment 1: can mites reduce lytic activity and its associated fitness costs

#### Lytic activity in response to manipulations of breeding conditions

**Figure 1:**
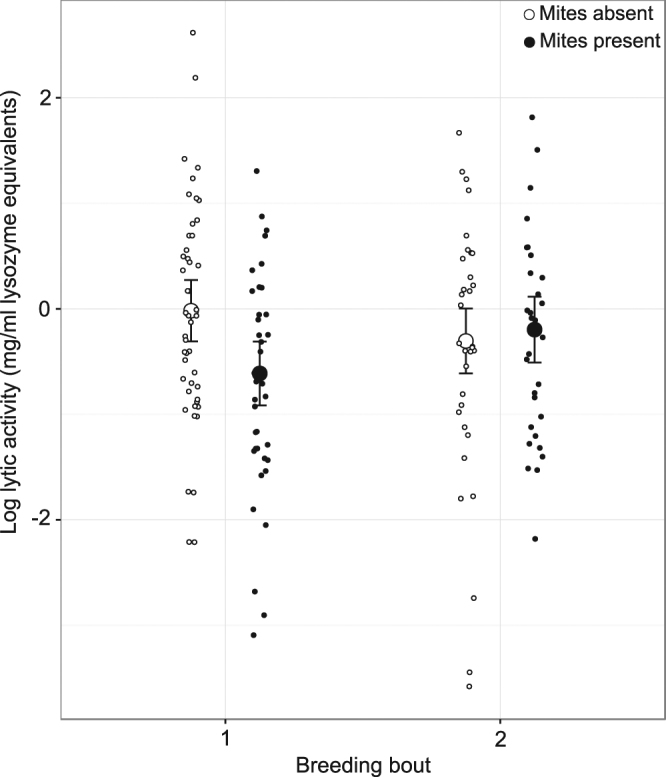
Female lytic activity (in mg/ml lysozyme equivalents) is lower in the presence of mites, in the first breeding bout, but not the second. Large circles represent least-square means recovered from the general linear mixed model in Table 1, vertical lines are standard errors from the same model. Small circles represent data points.

We found no significant interaction between mite presence and bacterial challenge on lytic activity (estimate = −0.25, *t*_77.64_ = −0.73, *p* = 0.47). However, our minimal adequate model showed that both mite and bacterial treatments were involved in statistically significant interactions with other covariates (Table 1). Females breeding with mites showed significantly lower lytic activity than females breeding without mites in the first breeding event (Fig. 1, Tukey post-hoc test: estimated difference = 0.59, *t*_132.11_ = 2.67, *p* = 0.042), but not in the second breeding event (estimated difference = −0.11, *t*_135.88_ = −0.422, *p* = 0.97).

**Table 1:** Summary of linear mixed model fitted by restricted maximum likelihood (REML) with female log lytic activity as response variable.

	Value	SE	DF	*t*-value	*p*-value
(Intercept)	−6.86	3.08	95.04	−2.22	**0**.03*
bacterial challenge	8.32	3.80	91.50	2.19	**0**.03*
mite presence	−0.59	0.22	132.11	−2.68	**0**.008**
female size	1.35	0.61	95.27	2.20	**0**.03*
breeding event	−0.29	0.21	66.22	−1.33	0.19
bacteria-dipping × female size	−1.64	0.76	92.05	−2.17	**0**.03*
mite presence × breeding event	0.70	0.32	70.46	2.21	**0**.03*

Model parameter estimates (value) and standard error (SE) are provided, as well as *t*-values and *p*-values for the estimates (*p*-values < 0.05 are statistically significant, in bold). Significance: *p < 0.05, **p < 0.01, ***p < 0.001.

**Figure 2:**
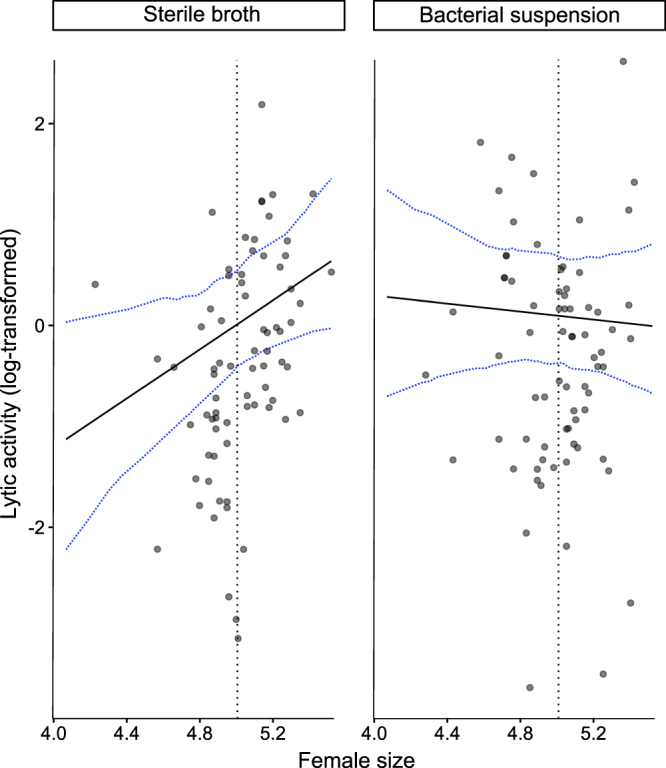
Relationship between lytic activity and female size depends on the microbial environment of the carcass. Circles show raw data. Black solid lines show predicted lytic activity values from a GLMM (Table 1) for a dummy data set of female size. Blue dotted lines show the 95% confidence interval for model predictions, derived with bootMer method. Black dotted lines indicate median female pronotum width.

We also found a significant interaction between the bacterial treatment and female size on lytic activity (Table 1). Figure 2 shows the predicted partial effects of female size and bacterial challenge (having averaged over all other effects): in carcasses that were not bacterially challenged, lytic activity was positively associated with female size. In bacterially-challenged carcasses, lytic activity was relatively high at all female sizes (but showed a non-significant trend of a decrease with female size; linear regression slope = −0.20, *F*_1,72_ = 0.16, *p* = 0.69). Taken together, the evidence suggests that smaller females increase lytic activity in response to a bacterial challenge, but larger females show high lytic activity regardless of bacterial conditions on the carcass.

#### Survival

**Figure 3:**
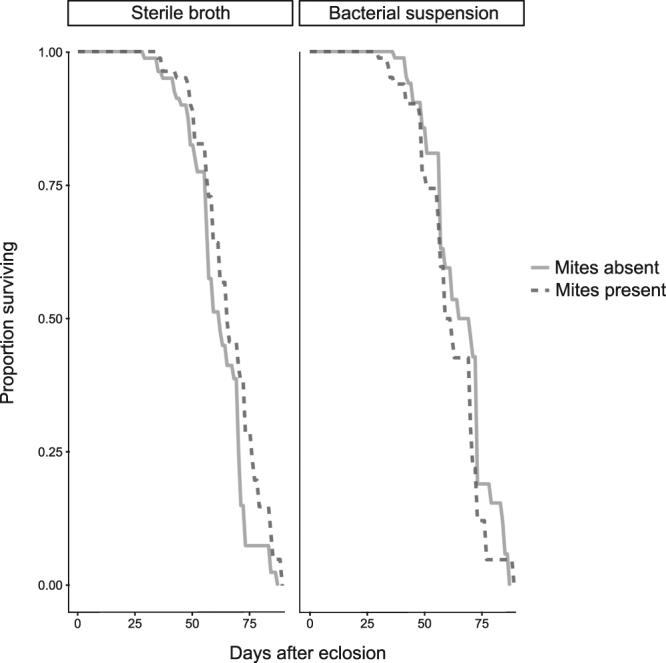
Survival curves for females across the four treatments. There is an interaction between mite and bacterial treatment, with females surviving slightly longer when they have bred alongside mites, on carcasses dipped in sterile nutrient broth.

We found a significant interaction between the effects of presence of mites and the bacterial treatment on female survival: for females that bred on control-dipped carcasses, the presence of mites had a positive effect on survival (Table 2; Fig. 3). For females that bred in bacteria-dipped carcasses, the presence of mites had no effect on survival. Female size had no effect on survival and did not interact significantly with treatment to influence survival (Table 2).

**Table 2:** Cox Proportional Hazards mixed effects model for female and male survival (i.e. days post-eclosion).

	Coefficient	SE	*z*-value	*p*-value
**Female**				
bacterial challenge	−0.34	0.23	−1.46	0.14
mite presence	−0.42	0.24	−1.74	0.08
bacterial challenge × mite presence	0.71	0.33	2.13	**0**.03*
**Male**				
mite presence	0.37	0.14	2.59	**0**.01*
female breeding bout	0.58	0.14	4.03	**0**.00055***
carcass mass	−0.11	0.04	−2.41	**0**.016*

Coefficient values are estimates of the effect of a factor on the risk of death; hence negative values indicate a positive effect on survival. Standard error (SE), *z*-values and *p*-values for the estimates are provided (*p*-values < 0.05 are statistically significant, in bold). Marginally non-significant: *p* < 0.1; significant: *p < 0.05, **p < 0.01, ***p < 0.001.

We recorded survival for males breeding with females during the female’s first two breeding bouts. As found previously^19^, male survival was negatively affected by the presence of mites (Table 2). Carcass-dipping treatment had no effect on male survival. Males whose partners were on their second breeding event had shorter lifespans than males paired with virgin females. Carcass mass had a significant positive effect on male survival (Table 2).

#### Reproductive output

Reproductive output (per brood) was measured in terms of brood size, brood mass, larval density (brood size at dispersal divided by carcass mass) and average larval mass. We also tested for differences in lifetime reproductive success (LRS) between treatments. Brood size, brood mass and larval density were not affected by mite presence or bacterial treatment (Table S2), nor was there an interaction between carcass treatments and breeding event for any of the brood measures Table S2 and Table 3, Figures S2–S5 in Supplementary Material). Overall, reproductive output was similar in the first two broods and started to decline in the third breeding event. Average larval mass (Table 3, Figure S5) was significantly lower in the second breeding event, when compared with the first breeding. Average larval mass increased significantly with carcass mass. Furthermore, an interaction between female size and mite treatment (Table 3, Figure S6) suggests a tendency for larger females to produce heavier larvae in the presence of mites. This interaction became marginally significant once three outliers were removed (model without outliers is shown in Table S3). For lifetime reproductive success (LRS), we found a significant interaction involving mite and bacterial treatment (Table 4), which became marginally significant when an outlier was removed (Table S4). Plotting this interaction suggests a similar pattern as the one found for female survival: females on carcasses without bacterial challenge tended to have slightly higher LRS when breeding alongside mites; females on bacterially challenged carcasses showed a tendency for lower LRS when breeding alongside mites (Figure S7).

**Table 3:** Summary of linear mixed models for average larval mass, fitted with REML.

Average larval mass	Value	SE	DF	*t-*value	*Χ* ^2^	*p*-value
(Intercept)	0.21	0.06	110.81	3.69		**3**.**5 × 10** ^**−4**^ ***
mite presence	−0.18	0.08	112.43	−2.12		**0**.04*
carcass mass	0.004	0.001	222.34	3.273		**0**.001**
breeding event 2	−0.01	0.004	165.27	−3.15		**0**.002*
breeding event 3	−0.008	0.005	180.80	−1.64		0.10
breeding event 4	−0.003	0.008	189.71	−0.32		0.75
breeding event (overall)	—	—	3	—	9.56	0.02*
female size	−0.02	0.01	110.42	−1.80		0.075
mite presence × female size	0.036	0.017	112.74	2.12		**0**.04*

The overall effect of breeding event was tested with likelihood ratio tests, for which we provide *Χ* ^2^ test statistics and *p*-values. Model parameter estimates (value) and standard error (SE) are provided, as well as *t*-values and *p*-values for the estimates (*p*-values < 0.05 are statistically significant, in bold). Marginally non-significant: *p* < 0.1; significant: *p < 0.05, **p < 0.01, ***p < 0.001.

**Table 4:** Summary of linear mixed model for lifetime reproductive success (LRS), fitted with REML.

LRS	Value	SE	DF	*t-*value	*p*-value
(Intercept)	27.716	5.291	3.720	5.238	**0**.008**
mite presence	5.997	4.519	109.790	1.327	0.19
bacterial challenge	4.672	4.446	107.860	1.051	0.29
mite presence × bacterial challenge	−12.947	6.246	108.94	−2.073	**0**.04*

Model parameter estimates (value) and standard error (SE) are provided, as well as *t*-values and *p*-values for the estimates (*p*-values < 0.05 are statistically significant, in bold). Significant: *p < 0.05, **p < 0.01, ***p < 0.001.

### Experiment 2: do mites alter bacterial communities on the carcass

First, we tested whether mites were reducing bacterial load, i.e. the number of bacterial cells, measured by qRT-PCR. The presence of mites had no effect on the bacterial load on carcasses (*t*_21_ = −0.70, *p* = 0.49). Carcasses dipped in bacterial suspension showed significantly higher bacterial load than carcasses dipped in sterile nutrient broth (*t*_21_ = 2.68, *p* = 0.01). There was no interaction between carcass-dipping and mite treatment on bacterial load (*F* = 0.63, *p* = 0.44).

**Figure 4:**
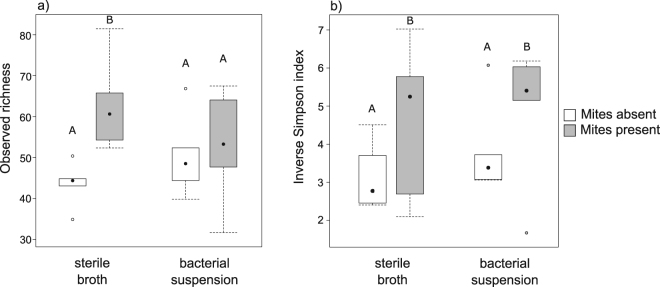
(**a**) Observed richness and (**b**) diversity (Inverse Simpson index) of bacterial communities on the carcasses across four treatments.

Next, using 16S rRNA compositional analysis we tested whether mites affect the carcass bacterial community by reducing the number of bacterial species (i.e. species richness) and/or their diversity. Values of observed richness and diversity for each sample are provided in Table S1 of the Supplementary Material. There was a significant interaction between the presence of mites and the carcass-dipping treatment on the number of observed OTUs: when carcasses were dipped in bacterial suspension, the presence of mites had no effect on observed richness (Tukey post-hoc test: *t* = −0.50, *p* = 0.62; Fig. 4); when carcasses were dipped in sterile nutrient broth, the presence of mites was associated with higher number of observed OTUs (*t* = −3.32, *p* = 0.003; Fig. 4a). Community diversity (calculated with the inverse Simpson index) increased in the presence of mites (*t* = 2.38, *p* = 0.03; Fig. 4b).

**Figure 5:**
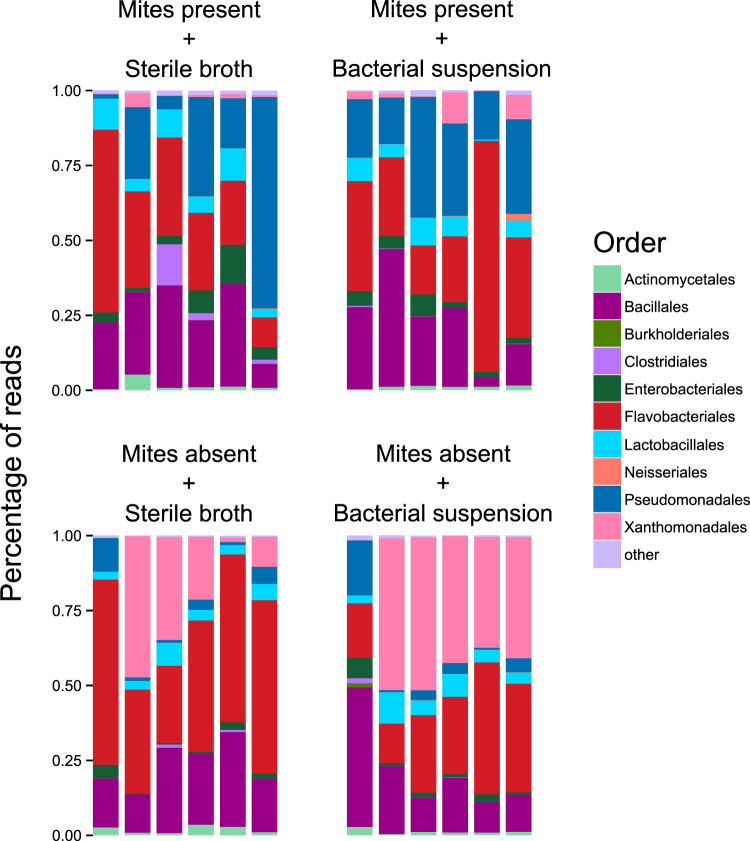
Composition of bacterial communities, classified to the order level, across the four treatments. Vertical axis depict the percentage of reads classified as belonging to a particular order. Each stacked bar corresponds to a single carcass.

Lastly, we tested whether differences could be found in the membership of the bacterial communities growing on carcasses, and which bacterial groups could be driving differences between treatments. We found that mite treatment had a significant effect on community composition (PERMANOVA: Pseudo-*F = *8.71, *p* = 0.001; Fig. 5), but there was no effect of dipping carcasses in a bacterial culture (Pseudo-*F* = 1.55, *p* = 0.19; Fig. 5). Differences between mite treatments cannot be attributed to different multivariate group dispersions, as these were found to be homogeneous (ANOVA: *F*_3_ = 0.81, *p* = 0.50). Reads assigned to Pseudomonadales were more frequent in carcasses with mites than carcasses without mites, whereas reads assigned to Xanthomonadales were in higher proportion of in mite-free carcasses (Fig. 5).

We then performed Indicator Species Analysis to statistically test which groups drive the differences between bacterial communities. Since carcass-dipping treatment had no effect on community composition, we grouped samples by mite treatment, and looked for OTUs which were significantly associated with mite presence and absence (Table 5). We found four OTUs significantly associated with the presence of mites on carcasses: two Pseudomonadales (*Acinetobacter* and one unclassified), one Flavobacteriales (*Myroides*) and one Enterobacteriales (unclassified). A single Xanthomonadales (*Wohlfahrtiimonas*) OTU was significantly associated with the absence of mites. The genus *Wohlfartiimonas* has two characterized species, both found in flies (Order Diptera) which feed on decaying organic matter^37,38^. All indicator OTUs have been previously found in association with beetle-prepared carcasses (such as *Acinetobacter* and *Myroides*)^30^ or gut bacterial communities of burying beetles (such as *Wohlfahrtiimonas*)^30,39^.

**Table 5:** Bacterial taxa associated with different treatments using Indicator Species Analysis.

*5Treatment*	*Order*	*Family*	*Genus*	*No of OTUs*	*IV*
**Mites present**	Pseudomonadales	Moraxellaceae	*Acinetobacter*	1	0.92
	Flavobacteriales	Flavobacteriaceae	*Myroides*	1	0.89
	Pseudomonadales	Moraxellaceae	Unclassified	1	0.89
	Enterobacteriales	Enterobacteriaceae	Unclassified	1	0.85
**Mites absent**	Xanthomonadales	Xanthomonadaceae	*Wohlfahrtiimonas*	1	0.96

Samples were grouped by mite treatment to identify bacterial OTUs associated with the presence versus absence of mites. Only significant (p < 0.05) taxa with Indicator Value (IV) > 0.85 are shown.

Overall, the bacterial communities in the present study were qualitatively similar in composition to the communities found previously in carcasses prepared by field-collected beetles in field-collected soil^30^, where the most abundant groups were also Bacillales, Flavobacteriales, Clostridiales and Pseudomonadales. Interestingly, despite the increased bacterial load in bacterially-challenged carcasses, we did not find any sequences belonging to *M*. *luteus* (Order Micrococcales), the bacterium used for the bacterial challenge treatment, in any of the carcass samples. This could be due to the high susceptibility of *M*. *luteus* to the antimicrobial exudates produced by *N*. *vespilloides* ^21,22^, or to an inability of this bacterium to colonize the carcass.

## Discussion

In this study we investigated whether burying beetles outsource some of the costs associated with antimicrobial defence of the carcass to mites, who may reduce microbes on the carcass as a by-product of grazing on the carrion’s surface. We found little support for this idea. Females did reduce their lytic activity in the presence of mites, but only in their first breeding event (Fig. 1). Furthermore, although mites had a weak but beneficial effect on female survival and lifetime reproductive success (Fig. 3), this was only the case when females bred on control carcasses that had been dipped in sterile nutrient broth. If mites clear the carcass of microbes, we would expect the benefits of breeding with mites to be clearer in the females breeding on carcasses exposed to a bacterial challenge. Instead, these females showed a weak tendency for shorter lifespan and life-time reproductive success than females breeding without mites. We also found that mites did not reduce bacterial load on the carcass, but that their presence was instead associated with higher bacterial richness and diversity.

**Figure 6:**
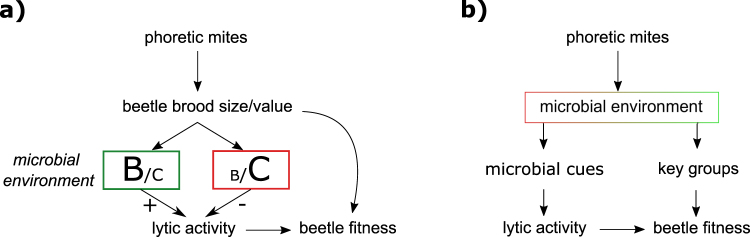
Scheme of how phoretic mites could affect social immune response (measured as lytic activity) and burying beetle fitness. (**a**) Mites directly affect brood size and/or value (positively or negatively), with direct fitness consequences for the burying beetle. Independently, the microbial environment alters the cost-benefit ratio of investment in lytic activity, and this is modulated accordingly, causing consequent changes in beetle fitness. (**b**) Mites directly affect the microbial environment, resulting in a change in bacterial cues that are used to stimulate lytic activity, with consequent changes in beetle fitness. In addition, or instead, mites influence the abundance of key bacterial groups and this has direct fitness consequences for the burying beetle.

Contrary to the expectation from the cleaning mutualism hypothesis, females only benefited from the presence of mites when carcasses were not bacterially-challenged. However, the reduction in female lytic activity during the first breeding event associated with mites was observed in both bacterially-challenged and unchallenged carcasses. We consider two possibilities to account for the observed reduction in lytic activity. The first, outlined in Fig. 6a, is that mites decrease the size or value of the current brood, which could lead females to reduce their investment in the first brood, in anticipation of a more successful second breeding attempt. We found no evidence to support this suggestion. Neither brood size nor brood value (i.e. brood mass, average larval mass, larval density) decreased in the presence of mites, just as we found previously when we allowed males to desert the brood after carcass preparation^14^.

An alternative possibility is that beetles are instead modulating their lytic activity in response to changes in the bacterial community on the carcass (Fig. 6b). The main active component of burying beetle anal exudates is an insect lysozyme^40,41^. Due to the absence of a lipopolysaccharide layer protecting the cell wall, Gram-positive bacteria are more sensitive to the action of lysozyme than their Gram-negative counterparts^42^. The groups of bacteria showing the largest differences in relation to mites were Pseudomonadales (abundant in the presence of mites) and Xanthomonadales (abundant in the absence of mites) (Fig. 5). These bacterial groups are both Gram negative, and hence less likely to be affected by lysozyme. Our results suggest that the observed changes in bacterial communities are not caused by changes in lytic activity that are provoked by mites interacting directly with beetles. Instead, our data suggests that the mites themselves are changing bacterial communities on the carcass and this is causing a change in the beetle’s lytic activity. For example, by increasing the abundance of Gram negative, lysozyme-resistant groups such as Pseudomonadales, mites may decrease the efficiency of lysozyme as a strategy for bacterial manipulation. This would imply that beetles regulate their lytic activity in relation to bacterial richness and diversity, as well as abundance (as shown in Cotter *et al*. 2010^21^). Further work is needed to understand the functional significance of the observed changes in bacterial community composition.

If the effect of mites on lytic activity is indeed a consequence of altered bacterial cues, why is this effect only observed in first-time breeders? One possibility is that younger individuals (first-time breeders) are more sensitive to environmental cues than older individuals because younger individuals have more residual reproductive value. There could therefore be stronger selection for young first-time breeders to adjust their phenotype to environmental conditions^43,44^. Further studies are needed to explore age-dependent plasticity in the antimicrobial defences of burying beetles.

Previous work has also shown that lytic activity increases when females bred in bacteria-dipped carcasses^21^, yet in the current study the effect was strongly size-dependent. We found that smaller females up-regulated lytic activity in response to a bacterial challenge, but larger females did not, showing consistently high lytic activity across environments instead. Contrary to Cotter *et al*. (2010)^21^, we did not find a clear fitness cost associated with up-regulating lytic activity, as the microbial challenge did not result in a shorter lifespan, nor decreased lifetime reproductive success. These results were not caused by variation in female size. Nor did bacterial treatment have any effect on most measures of reproductive output. The contrasting findings are instead more likely explained by a difference in the experimental protocol. In the study of Cotter and colleagues, males were removed after 24 h, before carcass preparation was complete. Here we allowed males to be present during carcass preparation, and to share the costs of carcass preparation with the female^19^. Our results suggest that fitness costs of up-regulating lytic activity are conditional on other energetic requirements, which may be greater when females perform pre- and post-hatching care by themselves. Similar context-dependent costs have been found for personal immunity in bumblebees^45^ and house-sparrows^46^.

We note that many of our analyses (e.g. lytic activity, larval mass and LRS; Tables 1, 3 and 4) indicate statistically significant interaction effects with *p*-values close to the cut-off of 0.05 for significance. Given the recent discussion of the ‘replication crisis’ in scientific research^47^ and the contrasts between our results and those of similar studies (as discussed above), additional studies of these effects would be particularly useful in determining whether these interactions stand up to further scrutiny.

## Concluding remarks

We have not found evidence demonstrating the existence of a by-product cleaning mutualism between burying beetles and mites. On the one hand we found that the mites are associated with a reduction in lytic activity and a tendency for increased fitness in female burying beetles. Yet these benefits are weak and most likely to be gained when females are breeding alone, and for the first time, and when the carcass is not bacterially-enriched, which contradicts expectations for a cleaning mutualism. Our results suggest that the combination of mites and bacterial challenge has negative effects on female fitness. We have also confirmed previous results that mites have detrimental effects on male burying beetles. Thus, like many interspecific interactions on the parasite-mutualism continuum, the outcome of the burying beetle-mite interaction is context-dependent^2^, fluctuating from parasitic to commensal to mutualistic according to which family member is involved and the wider ecological conditions. On the other hand, although we found that mites change the bacterial communities on the carcass, we do not yet know the functional significance of these changes from the beetle’s perspective, nor that they directly caused the reduction in the lytic activity of the beetle’s anal exudates. These changes might arise simply as a consequence of mites foraging on carrion, and may be selectively neutral from the beetle’s perspective. An alternative possibility is that the mite-induced increase in bacterial richness and diversity on the carcass promotes resistance to colonization by harmful microbes, but this remains to be tested in future work.

## Electronic supplementary material

Supplementary Material & Methods

## References

[1] Thompson JN (1988). Variation in interspecific interactions. Annu. Rev. Ecol. Syst..

[2] Bronstein JL (1994). Conditional outcomes in mutualistic interactions. Trends Ecol. Evol..

[3] Thompson JNC (2005). The Geographic Mosaic of Coevolutionary Arms Races. Curr. Biol..

[4] Moller AP, Christe P, Lux E (1999). Parasitism, Host Immune Function, and Sexual Selection. Q. Rev. Biol..

[5] Barber I, Hoare D, Krause J (2000). Effects of parasites on fish behaviour: a review and evolutionary perspective. Rev. Fish Biol. Fish..

[6] Sheldon BC, Verhulst S (1996). Ecological immunology: costly parasite defences and trade-offs in evolutionary ecology. Trends Ecol. Evol..

[7] Richner, H., Oppliger, A. & Christe, P. Effect of an ectoparasite on reproduction in great tits. *J*. *Anim*. *Ecol*. 703–710 (1993).

[8] Gallizzi K, Alloitteau O, Harrang E, Richner H (2008). Fleas, parental care, and transgenerational effects on tick load in the great tit. Behav. Ecol..

[9] Bristow CM (1983). Treehoppers transfer parental care to ants: A new benefit of mutualism. Science (80-.)..

[10] Pukowski E (1933). Ecological Investigation of Necrophorus F. - Ökologische untersuchungen an necrophorus F. Zoomorphology.

[11] Scott MP (1998). The Ecology and Behavior of Burying Beetles. Annu. Rev. Entomol..

[12] Cotter SC, Kilner RM (2010). Sexual division of antibacterial resource defence in breeding burying beetles, Nicrophorus vespilloides. J. Anim. Ecol..

[13] Pellissier Scott M, Traniello JFA (1990). Behavioural and ecological correlates of male and female parental care and reproductive success in burying beetles (Nicrophorus spp.). Anim. Behav..

[14] De Gasperin O, Duarte A, Kilner RM (2015). Interspecific interactions explain variation in the duration of paternal care in the burying beetle. Anim. Behav..

[15] Schwarz HH, Müller JK (1992). The dispersal behaviour of the phoretic mite Poecilochirus carabi (Mesostigmata, Parasitidae): adaptation to the breeding biology of its carrier Necrophorus vespilloides (Coleoptera, Silphidae). Oecologia.

[16] Schwarz HH, Starrach M, Koulianos S (1998). Host specificity and permanence of associations between mesostigmatic mites (Acari: Anactinotrichida) and burying beetles (Coleoptera: Silphidae: Nicrophorus). J. Nat. Hist..

[17] Wilson DS, Knollenberg WG (1987). Adaptive indirect effects: the fitness of burying beetles with and without their phoretic mites. Evol. Ecol..

[18] Schwarz HH, Koulianos S (1998). When to leave the brood chamber? Routes of dispersal in mites associated with burying beetles. Exp. Appl. Acarol..

[19] De Gasperin O, Kilner RM (2015). Interspecific interactions change the outcome of sexual conflict over prehatching parental investment in the burying beetle Nicrophorus vespilloides. Ecol. Evol..

[20] Cotter SC, Kilner RM (2010). Personal immunity versus social immunity. Behav. Ecol..

[21] Cotter SC, Topham E, Price AJP, Kilner RM (2010). Fitness costs associated with mounting a social immune response. Ecol. Lett..

[22] Arce AN, Johnston PR, Smiseth PT, Rozen DE (2012). Mechanisms and fitness effects of antibacterial defences in a carrion beetle. J. Evol. Biol..

[23] Lam K, Thu K, Tsang M, Moore M, Gries G (2009). Bacteria on housefly eggs, Musca domestica, suppress fungal growth in chicken manure through nutrient depletion or antifungal metabolites. Naturwissenschaften.

[24] Barnes KM, Gennard DE, Dixon RA (2010). An assessment of the antibacterial activity in larval excretion/secretion of four species of insects recorded in association with corpses, using Lucilia sericata Meigen as the marker species. Bull. Entomol. Res..

[25] Cotter SC, Littlefair JE, Grantham PJ, Kilner RM (2013). A direct physiological trade-off between personal and social immunity. J. Anim. Ecol..

[26] Bates D, Mächler M, Bolker B, Walker S (2015). Fitting Linear Mixed-Effects Models Using {lme4}. J. Stat. Softw.

[27] Kuznetsova, A., Bruun Brockhoff, P. & Haubo Bojesen Christensen, R. lmerTest: Tests in Linear Mixed Effects Models. (2016).

[28] Therneau, T. M. Coxme: Mixed Effects Cox Models. (2015).

[29] Zuur, A. F., Ieno, E. N., Walker, N. J., Saveliev, A. A. & Smith, G. M. In Mixed effects models and extensions in Ecology with R 101–142, 10.1007/978-0-387-87458-6 (Springer, 2009).

[30] Duarte, A., Welch, M., Swannack, C., Wagner, J. & Kilner, R. M. Strategies for managing rival bacterial communities: lessons from burying beetles. *J*. *Anim*. *Ecol*. (2017). 10.1111/1365-2656.12725 PMC5836980 28682460

[31] Schloss PD (2009). Introducing mothur: Open-Source, Platform-Independent, Community-Supported Software for Describing and Comparing Microbial Communities. Appl. Environ. Microbiol..

[32] Kozich JJ, Westcott SL, Baxter NT, Highlander SK, Schloss PD (2013). Development of a Dual-Index Sequencing Strategy and Curation Pipeline for Analyzing Amplicon Sequence Data on the MiSeq Illumina Sequencing Platform. Appl. Environ. Microbiol..

[33] Schloss PD, Westcott SL (2011). Assessing and Improving Methods Used in Operational Taxonomic Unit-Based Approaches for 16S rRNA Gene Sequence Analysis. Appl. Environ. Microbiol..

[34] Oksanen, J. *et al*. Vegan: Community Ecology Package. (2015).

[35] Cáceres MD, Legendre P (2009). Associations between species and groups of sites: indices and statistical inference. Ecology.

[36] Dufrêne M, Legendre P (1997). Species assemblages and indicator species:the need for a flexible asymmetrical approach. Ecol. Monogr..

[37] Tóth EM (2008). Wohlfahrtiimonas chitiniclastica gen. nov., sp. nov., a new gammaproteobacterium isolated from Wohlfahrtia magnifica (Diptera: Sarcophagidae). Int. J. Syst. Evol. Microbiol..

[38] Lee JK (2014). Wohlfahrtiimonas larvae sp. nov., isolated from the larval gut of Hermetia illucens (Diptera: Stratiomyidae). Antonie Van Leeuwenhoek.

[39] Kaltenpoth M, Steiger S (2014). Unearthing carrion beetles’ microbiome: Characterization of bacterial and fungal hindgut communities across the Silphidae. Mol. Ecol..

[40] Palmer, W. J. *et al*. A gene associated with social immunity in the burying beetle Nicrophorus vespilloides. *Proc*. *R*. *Soc*. *B Biol*. *Sci*. **283** (2016). 10.1098/rspb.2015.2733 PMC4795035 26817769

[41] Jacobs CGC (2016). Sex, offspring and carcass determine antimicrobial peptide expression in the burying beetle. Sci. Rep..

[42] Ibrahim HR, Kato A, Kobayashi K (1991). Antimicrobial effects of lysozyme against gram-negative bacteria due to covalent binding of palmitic acid. J. Agric. Food Chem..

[43] Fischer B, van Doorn GS, Dieckmann U, Taborsky B (2014). The Evolution of Age-Dependent Plasticity. Am. Nat..

[44] Fawcett TW, Frankenhuis WE (2015). Adaptive explanations for sensitive windows in development. Front. Zool..

[45] Moret Y, Schmid-Hempel P (2000). Survival for Immunity: The Price of Immune System Activation for Bumblebee Workers. Science (80-.).

[46] Bonneaud C (2003). Assessing the cost of mounting an immune response. Am. Nat..

[47] Ioannidis, J. P. A. Why Most Published Research Findings Are False. P*LOS Med*. **2** (2005). 10.1371/journal.pmed.0020124 PMC1182327 16060722

