# Supplementary material for: No evidence of a cleaning mutualism between burying beetles and their phoretic mites
Source: Sci Rep. 2017 Oct 23;7:13838. doi: 10.1038/s41598-017-14201-6 (PMC5653765; doi:10.1038/s41598-017-14201-6)
Supplement: Supplementary file 1 — Supplementary Material & Methods [file 41598_2017_14201_MOESM1_ESM.doc]

**No evidence of a cleaning mutualism between burying beetles and their phoretic mites**

Ana Duarte, Sheena C. Cotter, Ornela De Gasperin, Thomas M. Houslay, Giuseppe Boncoraglio, Martin Welch, Rebecca M. Kilner

### Supplementary Methods

### Quantitative real-time PCR

We chose a set of universal primers for the 16S rRNA gene. The primers have been reported to target 93.6% of all bacterial 16S rRNA sequences published and to be capable of detecting fewer than 100 copy numbers of the 16S gene (Cliffor*d et a*l., 2012).

A standard dilution series was created using genomic DNA from *Pseudomonas aeruginosa*. DNA was quantified with Qubit Fluorometer. DNA concentration of the standard solutions ranged from 14.2 ng/µl to 1.42 x10^-5^ ng/ µl.

We assessed via standard PCR which dilution of the samples’ DNA to use for quantitative PCR. Samples amplified successfully either with 10-fold or 100-fold dilutions. In each well of a 96-well plate, 2.5 µl of template were added to 10 µl of Fast SYBR® Green Master Mix, 1.25 µl of both primers (10 umol), and 5 µl of Milli-Q ultrapure filter-sterilized water, resulting in a final reaction volume of 20 µl. Quantitative PCR was performed with the 7300 Real-Time PCR System from Applied Biosystems®. The samples, standard curve, a positive control of unquantified *P. aeruginosa* DNA and a no-template control were all run in triplicate in the same 96-well plate. We followed cycle conditions advised for Fast SYBR® Green Master Mix: 95 °C for 20 seconds, then 40 cycles of 95 °C for 3 seconds and 60 °C for 30 seconds.

### Estimating bacterial DNA concentration

There is a linear relationship between the threshold cycle (C_T_) and the log concentration of DNA in the template. From the standard dilution series of known DNA quantities we calculated the slope and intercept of this linear equation. We then estimated the concentration of bacterial DNA present in samples, based on average C_T_ values obtained for each sample, and multiplying by the dilution factors used.

**Quality filtering of Illumina sequences**

Sequences containing > 250bp and more than 6 homopolymers were removed. To reduce sequence variation introduced by sequencing errors, sequences were trimmed with PRINSEQ-lite v.0.20.3 by 20 bp at the beginning of the forward reads and by 10 bp at the end of the reverse reads. This was necessary due to poor sequence quality at the end of the reads. The remaining sequences were aligned to the SILVA release 119 reference alignment, trimmed to the V3 region of the 16S rRNA-encoding gene. A post alignment screening was conducted and sequences with a search score below 80% and a similarity to the template sequences below 90% were excluded. Sequences were further de-noised during the pre-clustering steps and sequences with a difference of just 2 nucleotides were clustered together. Chimeric sequences were removed using the UCHIME algorithm (Edga*r et a*l., 2011) within MOTHUR. Sequences represented by fewer than 10 copies were excluded from further analysis. Those sequences were most likely left over low quality sequences escaping the quality filtering.

**Supplementary Tables and Figures**

**Table S1. Observed richness and diversity (Inverse Simpson index; average and 95% confidence intervals) of bacterial communities on sampled carcasses across the four treatments.**

| **Sample name** | **Observed richness** | **Inverse Simpson index (avg)** | **Inverse Simpson (lci)** | **Inverse Simpson (hci)** | **Microbial treatment** | **Mite treatment** |
| --- | --- | --- | --- | --- | --- | --- |
| CR2_MT190B | 66.84 | 6.07 | 5.92 | 6.23 | bacteria | absent |
| CR2_MT200B | 52.37 | 3.06 | 2.99 | 3.13 | bacteria | absent |
| CR2_MT210B | 50.75 | 3.14 | 3.07 | 3.22 | bacteria | absent |
| CR2_MT220B | 39.81 | 3.72 | 3.65 | 3.81 | bacteria | absent |
| CR2_MT230B | 46.27 | 3.08 | 3.03 | 3.13 | bacteria | absent |
| CR2_MT240B | 44.38 | 3.62 | 3.56 | 3.69 | bacteria | absent |
| CR2_MT100C | 50.37 | 2.40 | 2.35 | 2.46 | sterile | absent |
| CR2_MT110C | 34.87 | 2.86 | 2.82 | 2.91 | sterile | absent |
| CR2_MT120C | 44.66 | 4.51 | 4.42 | 4.59 | sterile | absent |
| CR2_MT70C | 43.07 | 3.71 | 3.64 | 3.78 | sterile | absent |
| CR2_MT80C | 44.06 | 2.45 | 2.41 | 2.50 | sterile | absent |
| CR2_MT90C | 44.83 | 2.69 | 2.63 | 2.76 | sterile | absent |
| CR2_MT13MB | 47.68 | 5.61 | 5.50 | 5.73 | bacteria | present |
| CR2_MT14MB | 54.78 | 6.18 | 6.08 | 6.29 | bacteria | present |
| CR2_MT15MB | 67.47 | 5.15 | 5.01 | 5.30 | bacteria | present |
| CR2_MT16MB | 51.76 | 6.03 | 5.91 | 6.16 | bacteria | present |
| CR2_MT17MB | 31.66 | 1.67 | 1.64 | 1.70 | bacteria | present |
| CR2_MT18MB | 64.05 | 5.20 | 5.08 | 5.33 | bacteria | present |
| CR2_MT1MC | 52.34 | 2.69 | 2.63 | 2.76 | sterile | present |
| CR2_MT2MC | 54.26 | 5.62 | 5.50 | 5.73 | sterile | present |
| CR2_MT3MC | 81.47 | 4.88 | 4.78 | 4.98 | sterile | present |
| CR2_MT4MC | 65.78 | 5.78 | 5.64 | 5.92 | sterile | present |
| CR2_MT5MC | 61.69 | 7.02 | 6.92 | 7.13 | sterile | present |
| CR2_MT6MC | 59.57 | 2.09 | 2.05 | 2.14 | sterile | present |

**Table S2**. Summary of linear mixed models for reproductive output measures, fitted with REML. The overall effect of breeding event was tested with likelihood ratio tests, for which we provide *Χ*^2^ test statistics and *p*-values. Model parameter estimates (value) and standard error (SE) are provided, as well as *t*-values and *p*-values for the estimates (*p*-values < 0.05 are statistically significant, in bold).

|  | **Value** | **SE** | **DF** | ***t-*value** | ***Χ*^2^** | ***p*-value** |
| --- | --- | --- | --- | --- | --- | --- |
| **Brood size** |  |  |  |  |  |  |
| (Intercept) | 11.09 | 1.39 | 2.8 | 7.95 |  | **0.006 **** |
| breeding event 2 | 1.39 | 0.94 | 314.2 | 1.47 |  | 0.14 |
| breeding event 3 | -3.79 | 1.02 | 334.5 | -3.70 |  | **0.0002 ***** |
| breeding event 4 | -8.65 | 1.35 | 369.2 | -6.43 |  | **<0.0001 ***** |
| breeding event (overall) | - | - | 3 | - | 60.304 | **<0.0001 ***** |
| **Brood mass** |  |  |  |  |  |  |
| (Intercept) | 1.54 | 0.16 | 2.9 | 9.567 |  | **0.003 **** |
| breeding event 2 | 0.04 | 0.11 | 314.4 | 0.36 |  | 0.72 |
| breeding event 3 | -0.59 | 0.12 | 335.30 | -4.80 |  | **<0.0001 ***** |
| breeding event 4 | -1.17 | 0.16 | 372.1 | -7.23 |  | **<0.0001 ***** |
| breeding event (overall) | - | - | 3 | - | 25.74 | **<0.0001 ***** |
| **Larval density** |  |  |  |  |  |  |
| (Intercept) | 1.04 | 0.13 | 2.80 | 8.00 |  | **0.005 **** |
| breeding event 2 | 0.15 | 0.09 | 311.8 | 1.59 |  | 0.11 |
| breeding event 3 | -0.32 | 0.10 | 332.5 | -3.24 |  | **0.0013 **** |
| breeding event 4 | -0.82 | 0.13 | 368.9 | -6.24 |  | **<0.0001 ***** |
| breeding event (overall) | - | - | 3 | - | 21.46 | **<0.0001 ***** |

Marginally non-significant: *p* < 0.1 . ; significant: *p* < 0.05 *, *p* < 0.01 **, *p* < 0.001 ***

**Table S3. Summary of linear mixed models for average larval mass, fitted with REML, after removal of three outliers. The model summary when outliers are included can be found in Table 4 in the main text.**

| **Average larval mass** | **Value** | **SE** | **DF** | ***t-*value** | ***p*-value** |
| --- | --- | --- | --- | --- | --- |
| (Intercept) | 0.220 | 0.060 | 109.900 | 3.878 | **< 0.001***** |
| bacterial challenge | -0.007 | 0.006 | 96.240 | -1.161 | 0.249 |
| mite presence | -0.161 | 0.083 | 107.000 | -1.951 | 0.054 |
| carcass mass | 0.004 | 0.001 | 283.200 | 3.919 | **< 0.001 ***** |
| breeding event 2 | -0.011 | 0.004 | 215.300 | -2.839 | **0.005** |
| breeding event 3 | -0.007 | 0.005 | 233.700 | -1.589 | 0.113 |
| breeding event 4 | -0.001 | 0.007 | 240.700 | -0.104 | 0.917 |
| female size | -0.023 | 0.011 | 109.600 | -1.987 | 0.049 |
| mite presence × bacterial challenge | 0.015 | 0.008 | 99.950 | 1.829 | 0.070 |
| mite presence × female size | 0.030 | 0.016 | 107.700 | 1.817 | 0.072 |

**Table S4.** Summary of linear mixed model for lifetime reproductive success (LRS), fitted with REML, after removal of one outlier. The model summary when outliers are included can be found in Table 5 in the main text.

| **LRS** | **Value** | **SE** | **DF** | ***t-*value** | ***p*-value** |
| --- | --- | --- | --- | --- | --- |
| (Intercept) | 27.53 | 4.737 | 3.940 | 5.811 | **0.005 **** |
| mite presence | 6.239 | 4.212 | 108.59 | 1.481 | 0.14 |
| bacterial challenge | 2.636 | 4.173 | 107.62 | 0.632 | 0.53 |
| mite presence × bacterial challenge | -11.042 | 5.841 | 108.18 | -1.890 | 0.061 . |

**Supplementary Figures**

**Figure S1.** Number of dispersing larvae in pilot experiment, where females were allowed to mate with males for 24h, then placed on a carcass. Females performed pre and post-hatching care on their own. Carcasses were either dipped in a sterile nutrient broth (control-dipped) or in a bacterial broth (bacteria-dipped). Carcasses were either kept mite-free or 10 deutonymphs of *P. carabi* were added. *N* = 6 per treatment.


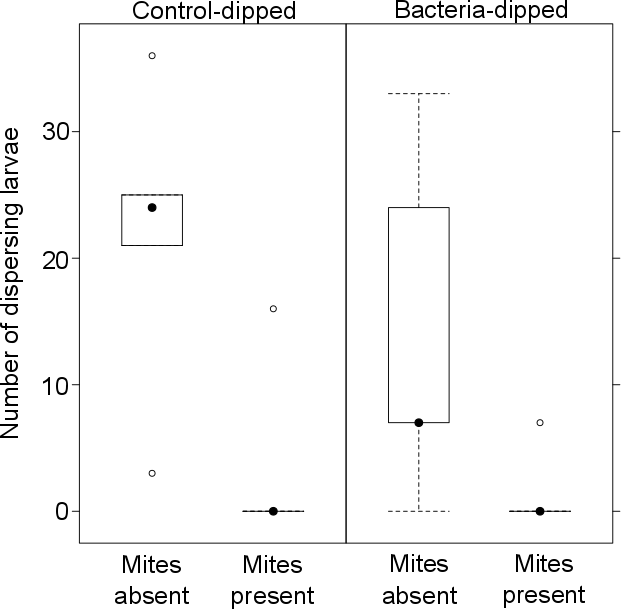


**Figure S2.** Brood size at dispersal across breeding bouts.

**
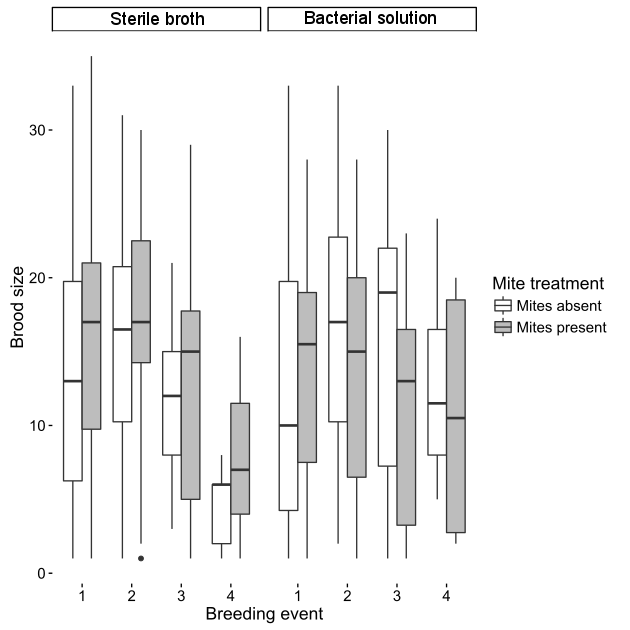
**

**Figure S3.** Brood mass at dispersal across breeding bouts.

**
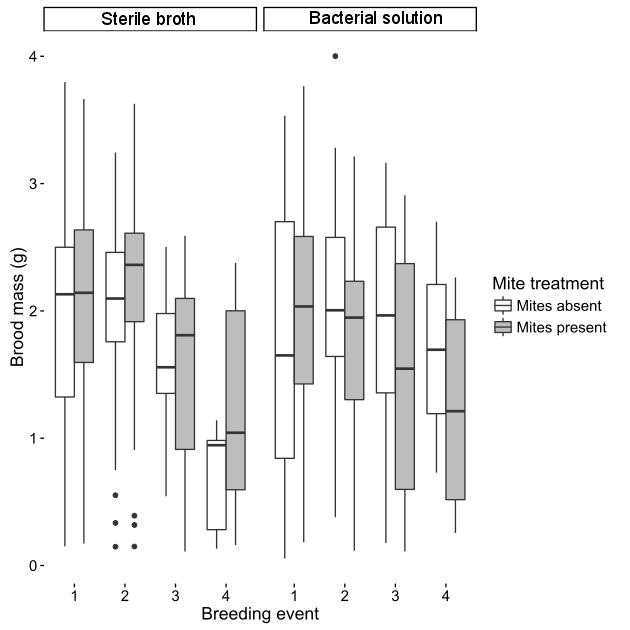
**

**Figure S4.** Larval density across breeding bouts.

**
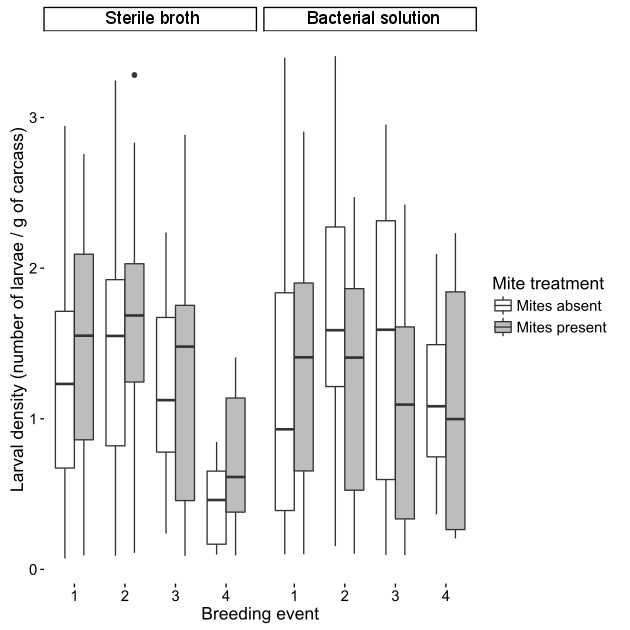
**

**Figure S5.** Average larval mass across breeding bouts.

**
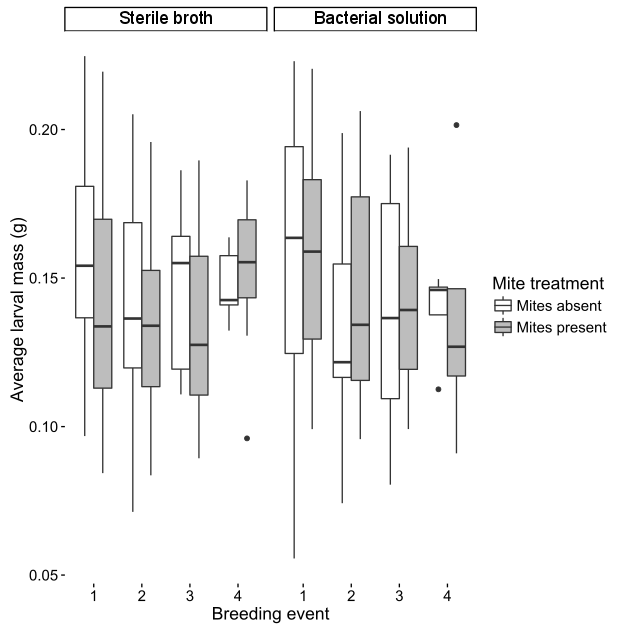
**

**Figure S6.** Interaction between mite treatment and female size for average larval mass. Blue lines indicate linear regressions of average larval mass on female pronotum width, with shaded areas indicating 95% confidence intervals. Data points encircled in red are outliers removed to check the robustness of the results. **
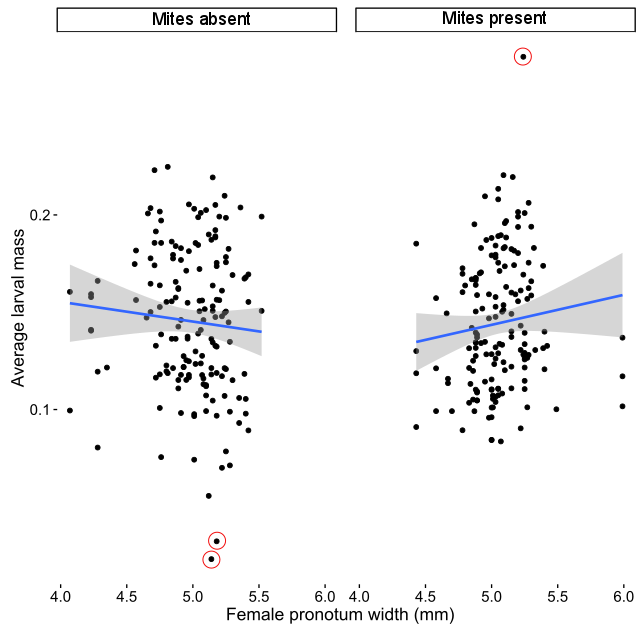
**

**Figure S7.** Interaction between mite treatment and microbial treatment for lifetime reproductive success. Data point encircled in red is an outlier removed to check the robustness of the results.

**
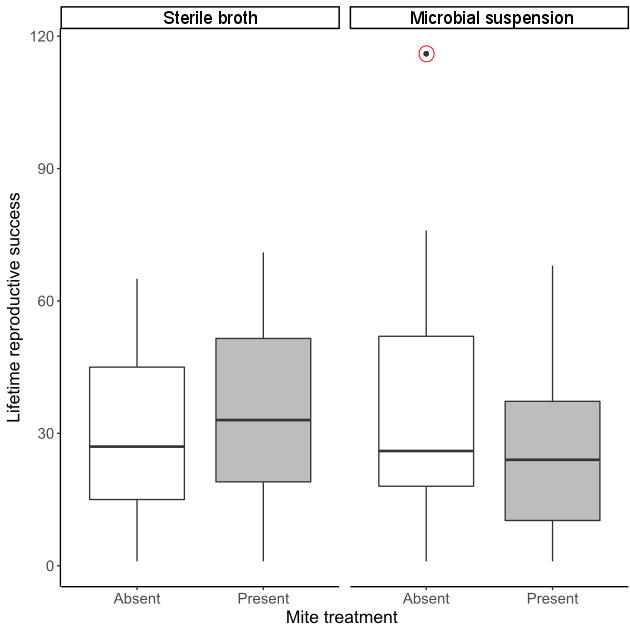
**
